# Supplementary material for: Allosteric regulation and crystallographic fragment screening of SARS-CoV-2 NSP15 endoribonuclease
Source: Nucleic Acids Res. 2023 Apr 28;51(10):5255–70. doi: 10.1093/nar/gkad314 (PMC10250223; doi:10.1093/nar/gkad314)
Supplement: gkad314_Supplemental_Files [file gkad314_supplemental_files.zip › Supplementary datasets.zip!/183/Refine_4/01-BUSTER/Cycle-3/shell.01/R_Rfree.html]

# Rxpct factors in resolution bins at cycle 0


|  |  |  |  |  |  |  |  |  |
| --- | --- | --- | --- | --- | --- | --- | --- | --- |
|  | **<d> (Å)**  Rxpct\_Work | Rxpct\_Free | Rxpct\_All | |  |  |  |  |
|  | 14.0455 | 0.3026 | 0.3348 | 0.3039 |  |
|  | 8.1092 | 0.2205 | 0.2585 | 0.2228 |  |
|  | 6.2814 | 0.2504 | 0.2655 | 0.2512 |  |
|  | 5.3087 | 0.2261 | 0.2572 | 0.2276 |  |
|  | 4.6818 | 0.2006 | 0.2136 | 0.2014 |  |
|  | 4.2349 | 0.2067 | 0.1848 | 0.2056 |  |
|  | 3.8955 | 0.2069 | 0.2256 | 0.2078 |  |
|  | 3.6265 | 0.2174 | 0.2679 | 0.2200 |  |
|  | 3.4065 | 0.2129 | 0.2301 | 0.2138 |  |
|  | 3.2223 | 0.2060 | 0.2173 | 0.2067 |  |
|  | 3.0650 | 0.2031 | 0.1921 | 0.2027 |  |
|  | 2.9287 | 0.1963 | 0.1921 | 0.1961 |  |
|  | 2.8091 | 0.1960 | 0.2139 | 0.1968 |  |
|  | 2.7031 | 0.2020 | 0.2157 | 0.2028 |  |
|  | 2.6082 | 0.2102 | 0.2252 | 0.2110 |  |
|  | 2.5227 | 0.2093 | 0.2223 | 0.2101 |  |
|  | 2.4450 | 0.2115 | 0.2328 | 0.2125 |  |
|  | 2.3741 | 0.2245 | 0.2300 | 0.2248 |  |
|  | 2.3091 | 0.2463 | 0.2545 | 0.2467 |  |
|  | 2.2491 | 0.3101 | 0.3109 | 0.3101 |  |
|  | **Overall** | 0.2176 | 0.2315 | 0.2183 |  |

# Rxpct factors in resolution bins at cycle 1


|  |  |  |  |  |  |  |  |  |
| --- | --- | --- | --- | --- | --- | --- | --- | --- |
|  | **<d> (Å)**  Rxpct\_Work | Rxpct\_Free | Rxpct\_All | |  |  |  |  |
|  | 14.0455 | 0.3055 | 0.3410 | 0.3069 |  |
|  | 8.1092 | 0.2233 | 0.2593 | 0.2254 |  |
|  | 6.2814 | 0.2500 | 0.2648 | 0.2507 |  |
|  | 5.3087 | 0.2246 | 0.2583 | 0.2262 |  |
|  | 4.6818 | 0.1978 | 0.2129 | 0.1987 |  |
|  | 4.2349 | 0.2042 | 0.1839 | 0.2031 |  |
|  | 3.8955 | 0.2034 | 0.2256 | 0.2045 |  |
|  | 3.6265 | 0.2164 | 0.2647 | 0.2189 |  |
|  | 3.4065 | 0.2116 | 0.2283 | 0.2124 |  |
|  | 3.2223 | 0.2061 | 0.2155 | 0.2067 |  |
|  | 3.0650 | 0.2043 | 0.1953 | 0.2039 |  |
|  | 2.9287 | 0.1978 | 0.1907 | 0.1975 |  |
|  | 2.8091 | 0.1978 | 0.2147 | 0.1986 |  |
|  | 2.7031 | 0.2033 | 0.2179 | 0.2041 |  |
|  | 2.6082 | 0.2119 | 0.2275 | 0.2128 |  |
|  | 2.5227 | 0.2101 | 0.2220 | 0.2108 |  |
|  | 2.4450 | 0.2125 | 0.2349 | 0.2136 |  |
|  | 2.3741 | 0.2241 | 0.2310 | 0.2244 |  |
|  | 2.3091 | 0.2429 | 0.2513 | 0.2433 |  |
|  | 2.2491 | 0.3147 | 0.3141 | 0.3146 |  |
|  | **Overall** | 0.2173 | 0.2315 | 0.2180 |  |

# Rxpct factors in resolution bins at cycle 2


|  |  |  |  |  |  |  |  |  |
| --- | --- | --- | --- | --- | --- | --- | --- | --- |
|  | **<d> (Å)**  Rxpct\_Work | Rxpct\_Free | Rxpct\_All | |  |  |  |  |
|  | 14.0455 | 0.3053 | 0.3398 | 0.3067 |  |
|  | 8.1092 | 0.2234 | 0.2591 | 0.2255 |  |
|  | 6.2814 | 0.2494 | 0.2620 | 0.2500 |  |
|  | 5.3087 | 0.2238 | 0.2583 | 0.2254 |  |
|  | 4.6818 | 0.1974 | 0.2126 | 0.1982 |  |
|  | 4.2349 | 0.2042 | 0.1833 | 0.2031 |  |
|  | 3.8955 | 0.2030 | 0.2236 | 0.2040 |  |
|  | 3.6265 | 0.2162 | 0.2627 | 0.2186 |  |
|  | 3.4065 | 0.2113 | 0.2286 | 0.2123 |  |
|  | 3.2223 | 0.2037 | 0.2145 | 0.2043 |  |
|  | 3.0650 | 0.2023 | 0.1920 | 0.2019 |  |
|  | 2.9287 | 0.1946 | 0.1853 | 0.1942 |  |
|  | 2.8091 | 0.1947 | 0.2092 | 0.1954 |  |
|  | 2.7031 | 0.2002 | 0.2152 | 0.2010 |  |
|  | 2.6082 | 0.2089 | 0.2251 | 0.2098 |  |
|  | 2.5227 | 0.2067 | 0.2209 | 0.2075 |  |
|  | 2.4450 | 0.2095 | 0.2306 | 0.2105 |  |
|  | 2.3741 | 0.2241 | 0.2282 | 0.2243 |  |
|  | 2.3091 | 0.2430 | 0.2541 | 0.2434 |  |
|  | 2.2491 | 0.3130 | 0.3128 | 0.3130 |  |
|  | **Overall** | 0.2161 | 0.2299 | 0.2168 |  |

# Rxpct factors in resolution bins at cycle 3


|  |  |  |  |  |  |  |  |  |
| --- | --- | --- | --- | --- | --- | --- | --- | --- |
|  | **<d> (Å)**  Rxpct\_Work | Rxpct\_Free | Rxpct\_All | |  |  |  |  |
|  | 14.0455 | 0.3067 | 0.3370 | 0.3079 |  |
|  | 8.1092 | 0.2219 | 0.2659 | 0.2245 |  |
|  | 6.2814 | 0.2406 | 0.2579 | 0.2415 |  |
|  | 5.3087 | 0.2194 | 0.2617 | 0.2214 |  |
|  | 4.6818 | 0.1935 | 0.2107 | 0.1944 |  |
|  | 4.2349 | 0.2016 | 0.1879 | 0.2009 |  |
|  | 3.8955 | 0.2018 | 0.2248 | 0.2029 |  |
|  | 3.6265 | 0.2148 | 0.2632 | 0.2172 |  |
|  | 3.4065 | 0.2096 | 0.2289 | 0.2107 |  |
|  | 3.2223 | 0.2031 | 0.2148 | 0.2038 |  |
|  | 3.0650 | 0.1974 | 0.1893 | 0.1970 |  |
|  | 2.9287 | 0.1913 | 0.1880 | 0.1911 |  |
|  | 2.8091 | 0.1924 | 0.2004 | 0.1927 |  |
|  | 2.7031 | 0.1948 | 0.2116 | 0.1957 |  |
|  | 2.6082 | 0.2028 | 0.2206 | 0.2039 |  |
|  | 2.5227 | 0.1987 | 0.2158 | 0.1997 |  |
|  | 2.4450 | 0.1999 | 0.2198 | 0.2009 |  |
|  | 2.3741 | 0.2140 | 0.2198 | 0.2143 |  |
|  | 2.3091 | 0.2345 | 0.2488 | 0.2352 |  |
|  | 2.2491 | 0.3042 | 0.3127 | 0.3046 |  |
|  | **Overall** | 0.2125 | 0.2293 | 0.2134 |  |
